# Editorial: Advancements in translational models: bridging basic infection research and clinical applications

**DOI:** 10.3389/fmed.2025.1730195

**Published:** 2025-11-18

**Authors:** Artur Schmidtchen, Aftab Nadeem, Haris Mirza, Karl Wallblom, Deepak Bushan Raina, Oonagh Shannon, Jitka Petrlova, Manoj Puthia

**Affiliations:** 1 Division of Dermatology and Venereology, Department of Clinical Sciences, Lund University, Lund, Sweden; 2 Department of Biomedical Sciences, Copenhagen Wound Healing Center, Bispebjerg Hospital, University of Copenhagen, Copenhagen, Denmark; 3 Department of Molecular Biology and Umeå Centre for Microbial Research (UCMR), Umeå University, Umeå, Sweden; 4 Department of Immunobiology, Yale University School of Medicine, New Haven, CT, United States; 5 Department of Clinical Sciences Lund, Orthopedics, Faculty of Medicine, Lund University, Lund, Sweden; 6 Section for Oral Biology and Pathology, Faculty of Odontology, Malmö University, Malmö, Sweden; 7 Department of Biomedical Science, Faculty of Health and Society, Biofilms Research Centre for Biointerfaces, Malmö University, Malmö, Sweden

**Keywords:** translational model, precision medicine, infection, microbiology, *in vivo*

The field of infection research is at a transformative phase, where emerging infections, antimicrobial resistance, and technological advances necessitate the development of novel translational models capable of effectively bridging laboratory discoveries with clinical applications. Here, we examine significant developments and highlight key innovations and challenges in improving therapeutic development and precision medicine.

Microfluidic organ-on-chip systems have emerged as innovative tools that precisely recapitulate human tissue architecture and function with remarkable accuracy. For instance, Zhang et al. (1) successfully developed a biomimetic lung chip that integrates alveolar epithelium, endothelium, and immune cells under fluidic flow, enabling them to systematically study SARS-CoV-2 infection and its elicited immune responses. Furthermore, Nof et al. (2) introduced a multi-compartment airway-on-chip platform capable of simulating regional lung crosstalk and airflow-mediated viral transmission from the nose to pulmonary acini. These refined microfluidic platforms permit real-time monitoring of complex host-pathogen interactions and maintain the architectural and functional characteristics of native tissues, which offers human-relevant experimental conditions previously difficult with traditional methods (3).

High-throughput capabilities have been significantly improved, largely through the development of new platforms, such as the PREDICT96-ALI system developed by Fisher et al. (4). This system enables rapid screening of SARS-CoV-2 therapeutic interventions using human primary epithelial airway cells. The integration of advanced sensing and monitoring capabilities within these systems has expedited drug repurposing pipelines and therapeutic evaluation, positively impacting the pace of discovery in infectious disease research.

Three-dimensional (3D) organoid models have improved our understanding of infection dynamics by providing physiologically relevant environments that cannot be replicated by traditional 2D models. For example, human pluripotent stem cell-derived organoids, integrated with immune and vascular components, allowed for single-cell multi-omics analysis to elucidate SARS-CoV-2 pathogenesis (5). Similarly, advanced studies of neurotropic infections have been performed using brain organoids, which established culture systems specific for modeling CNS infections (6). Kim et al. (7) developed an innovative human lung organoid model combined with macrophages for long-term tuberculosis infection studies, which provided novel platforms for host-directed therapy development. The successful integration of diverse cell types and complex matrix components within these three-dimensional (3D) systems has yielded extraordinary fidelity in capturing human tissue responses to a wide array of infections.

Humanized animal models represent critical translational advancements; however, their direct implementation remains comparatively limited due to the progress in sophisticated *in vitro* systems. Nevertheless, hybrid approaches are becoming popular, such as Sutton et al. (8) highlighted the potential of combining organoid technologies with humanized animal components to create pathogen screening platforms. This integration highlights an exciting path forward, where future translational models can become more complete by combining the unique advantages of both *in vitro* and *in vivo* research.

While human-relevant *in vitro* systems are essential, animal models remain invaluable for testing pathophysiological hypotheses about infection endotypes—biologically coherent disease subtypes. These models allow researchers to carefully control variables like host genetics, microbiota, exposure dose and timing, and comorbidities—factors that cannot be easily controlled in human patients. Complementing these approaches, state-of-the-art mass spectrometry proteomics provides quantitative resolution of host-pathogen programs that define infection endotypes. Modern DIA and TMT workflows quantify thousands of proteins across cohorts, enabling identification of outcome-linked modules and endotype classifiers (9, 10). Advanced peptidomics methodologies comprehensively analyze the endogenous peptidome generated by proteolytic activity, addressing challenges in achieving precision medicine for distinct inflammatory disease subtypes. Recent progress in peptidomics analyses and computational tools (11, 12) has enabled large-scale peptidome analysis. A computational workflow leveraging peptide clustering to enhance data resolution and inter-sample comparability (13) extends peptidomics toward clinical translation, defining temporal peptide signatures that reflect protease activity and infection dynamics.

The development of hybrid computational-experimental systems is a significant innovation identified in recent studies. Researchers successfully integrated malaria-on-a-chip devices with advanced pharmacokinetic/pharmacodynamic (PK/PD) modeling (14). This approach allowed researchers to take what they learned in the laboratory and directly predict how treatments would work in living organisms. This represents the successful implementation of ‘digital twin' technology in infectious disease research, opening up exciting new possibilities for predicting treatment outcomes.

The combination of multiple research platforms has become a hallmark of modern translational models. Organoids can now recreate complex disease conditions by bringing together different tissue components, allowing organs to communicate with each other, and incorporating the critical interactions between hosts and their microbiomes (15). Additionally, researchers have developed the ability to test treatments on a much larger scale by using advanced genetic engineering techniques and carefully controlling oxygen levels. This has significantly improved the ability of these combined systems to translate laboratory findings into real-world medical applications. Building on these advances, 3D bioprinting technology has transformed the creation of tissue models tailored to individual patients. For instance, researchers have developed 3D-printed lung-on-a-chip models that can monitor bacterial infections in real time (16). These bioprinted systems are especially valuable for studying respiratory diseases as they can accurately mimic how infections behave in patients, providing a personalized approach to understand how diseases develop and how treatments might work for individuals. Research on respiratory infections has become highly sophisticated, with many significant studies focusing on SARS-CoV-2 and other important lung pathogens. Scientists have created platforms that can accurately model complex viral infections (17) and various bacterial infections by combining organ-on-chip technology with advanced cell culture techniques (18). These advanced models are particularly useful for studying how viruses and bacteria interact together during infections and for understanding how our immune systems respond to these challenges.

New brain organoid technologies are transforming brain infection research. For instance, advanced 2D and 3D brain models that include immune cells and realistic blood-brain barriers are being used (19). These sophisticated systems are specifically built to study brain infections caused by pathogens such as Zika and dengue viruses. These platforms have made it possible for researchers to investigate how pathogens invade the brain—something that was nearly impossible to study using traditional research methods.

Advanced intestinal models have greatly enhanced antimicrobial resistance research. For example, Kaden et al. (19) showed how 3D intestine-on-chip systems can accurately mimic what happens when the antifungal drug caspofungin is administered intravenously. Their work revealed how fungal colonies of Candida albicans alter their behavior and disease-causing patterns in these models, representing a significant step forward in understanding how drugs and pathogens interact under conditions that closely resemble the human body. Intestinal infection research has made major progress thanks to the development of human intestinal organoids that contain multiple cell types. Scientists have created innovative platforms specifically designed to study how bacteria and viruses interact in the gut with their hosts (20). This new approach has helped researchers uncover previously unknown ways that microbes can both live peacefully alongside us and cause disease in the intestinal environment.

Several studies within this Research Topic illustrate these translational advances. van der Plas et al. demonstrated how Pseudomonas aeruginosa elastase strategically evades host immune responses by degrading pro-inflammatory cytokines and chemokines, providing mechanistic insights into non-healing wound pathogenesis through complementary porcine wound models and *in vitro* assays. Their work reveals how bacterial proteases contribute to the persistence of chronic infections by actively suppressing inflammatory responses, offering new targets for therapeutic intervention. By combining animal models with molecular analysis, van der Plas et al. show us why some infections resist treatment—in this case, how bacteria actively suppress immune responses to survive. This work demonstrates the real value of hybrid approaches: they help us understand not just what happens during infection, but why standard drugs sometimes fail. Their insights open new possibilities for designing better *in vitro* systems that can reproduce these immune-evasion tactics, ultimately helping us develop more effective therapies before they reach patients.

Zhao et al. developed a predictive algorithm integrating the neutrophil-to-lymphocyte ratio and CRP-to-albumin ratio to identify hemodialysis patients at risk for catheter-related bloodstream infections. By leveraging readily available hematological parameters, their approach demonstrates efficient biomarker discovery without requiring expensive new diagnostic tests. Multicenter validation of this algorithm demonstrates a key principle in translational research where research findings become clinically useful when we connect laboratory discoveries with practical tools that help identify high-risk patients. This work illustrates how precision medicine advances through systematic interrogation of existing clinical data, often yielding greater clinical utility than novel molecular biomarkers alone. Such approaches strengthen the translational pipeline by identifying cost-effective solutions that improve patient outcomes in vulnerable populations.

Raissi-Dehkordi et al. comprehensively reviewed the therapeutic mechanisms of cold atmospheric plasma in wound healing, highlighting its multi-modal benefits, including antimicrobial effects, angiogenesis stimulation, and biofilm disruption across chronic and acute wounds. Their analysis of cellular and molecular mechanisms demonstrates how innovative physical therapies can bridge laboratory discoveries with clinical applications in wound care.

The key to making these advanced models truly useful in medicine is validating them against real patient data. Some studies have shown encouraging results, such as Zhang et al. (1) validated their lung chip model against COVID-19 disease responses, and Fisher et al. (4) showed that their high-throughput testing platform matched human treatment responses. Yang et al. conducted a randomized controlled trial to evaluate remote ischemic post-conditioning in laparoscopic hepatectomy, demonstrating the critical importance of rigorous clinical validation even when experimental models are promising. Their study illustrates how translational research must bridge the gap between preclinical findings and clinical reality through carefully designed human trials. Although preliminary animal data had shown protective effects, this work reveals that hepatic ischemia-reperfusion injury in complex surgical scenarios involves multifactorial mechanisms that cannot always be fully recapitulated in simplified experimental systems, highlighting the important role of well-designed clinical trials in determining true therapeutic benefit and patient outcomes. However, the comprehensive clinical validation of these models in real clinical settings remains a significant challenge.

Mu et al. investigated the complex neuroinflammatory mechanisms triggered by intestinal surgery, revealing how cytokine cascades and glial cell activation drive post-operative complications. By integrating advanced neuroimaging techniques—including high-resolution MRI and PET imaging—with mechanistic understanding of these inflammatory pathways, their work demonstrates how translational insights can guide the development of precision treatment strategies that combine pharmacological interventions with emerging neuromodulatory approaches. This multidisciplinary approach demonstrates how mechanistic insights translate into personalized treatment strategies. Their comprehensive analysis highlights that addressing complex surgical complications requires sophisticated translational models capable of capturing the full spectrum of host responses, ultimately shaping clinical practice in ways that improve patient recovery and outcomes.

Looking ahead, computational integration represents the next major advancement in translational modeling, although we are still in the early stages of fully implementing these technologies. Combining advanced ML algorithms with multi-omics analysis holds great promise for speeding up drug discovery and helping us better understand how viruses evolve. Additionally, researchers are developing sophisticated computer models that track how diseases spread through populations—a crucial advancement for translating laboratory discoveries and predicting how they will work in real-world medical applications.

Finally, Tavecchio et al. developed an innovative murine pressure ulcer model that integrates magnet-induced ischemic injury with bioluminescent Staphylococcus aureus infection, enabling real-time kinetic monitoring of infection progression and therapeutic efficacy through bioluminescence imaging. By combining a physiologically relevant wound model with a tracking system that illuminates bacterial burden *in vivo*, this approach addresses a critical limitation of traditional pressure ulcer animal models: the inability to visualize and quantify infection dynamics non-invasively over time. This advance demonstrates how integrating cutting-edge imaging technologies with established animal models can transcend the limitations of either approach alone, generating mechanistic insights into chronic wound infection pathogenesis that neither laboratory nor conventional animal studies could achieve independently. Furthermore, their model illustrates the hybrid translational approach discussed throughout this editorial—where sophisticated technology platforms enhance rather than replace traditional methodologies, ultimately strengthening the bridge between preclinical discovery and therapeutic development.

Despite these exciting technological advances, standardization remains a significant challenge across different research platforms. Many studies acknowledge the ongoing difficulties associated with creating consistent methods, scaling up approaches, and thorough validation of results. Therefore, developing strong, standardized protocols and clear regulatory guidelines for these rapidly evolving technologies is important for their widespread use in drug development and clinical practice. The limited clinical validation in current studies simply reflects that this field is still in its early stages of development. Substantial research investment is essential to unlock the full potential of these advancements.

In conclusion, these breakthrough advances stem from organ-on-chip platforms, 3D organoid technologies, and hybrid computational-experimental systems that combine cell culture methods, multi-omics analysis, and computational modeling to accelerate therapeutic discovery and precision medicine. Key achievements include enhanced physiological relevance, high-throughput screening capabilities, and successful modeling of complex pathogens from SARS-CoV-2 to tuberculosis and malaria, with hybrid systems representing a paradigm shift toward true translational applications. However, significant challenges remain in validation, standardization, and clinical implementation, requiring sustained investment in validation studies, standardized protocols, and regulatory frameworks to determine whether these platforms can fulfill their promise of bridging research discoveries with life-saving clinical interventions in the fight against infectious diseases.

This Research Topic presents six compelling studies that illustrate the translational potential of research models across diverse infection-related challenges. These works collectively demonstrate how advanced experimental approaches, rigorous clinical validation, innovative therapeutic strategies, and sophisticated monitoring technologies can be integrated to address fundamental questions in infection research while maintaining clear pathways to clinical application. The diversity of approaches—from molecular mechanisms of pathogen evasion to clinical prediction algorithms and novel therapeutic modalities—highlights the multifaceted nature of translational infection research and the continued need for interdisciplinary collaboration to bridge the gap between laboratory discoveries and patient care.
